# Effect of anti-obesity drug orlistat (Xenical™) on liver and kidney function in male rats fed a high-fat diet

**DOI:** 10.12688/f1000research.174090.1

**Published:** 2026-01-29

**Authors:** Sura M. Alkadhimy, Asmaa M. Neamah, Mohammed Y.I. Al-Hamadani, Ahmed S. Jarad, Mokhtar I Yousef

**Affiliations:** 1 Department of Biology, College of Science, University of Karbala, Karbala, Karbala, 00964, Iraq; 2 Department of Environment, College of Science, University of Al-Qadisiyah, Al-Qadisiyah, Al-Qadisiyah, 00964, Iraq; 3 Department of Biology, University of Fallujah, Al-Fallujah, Al Anbar Governorate, 00964, Iraq; 4 Department of Pathology and Poultry Disease, College of Veterinary Medicine, University of Fallujah, Al-Fallujah, Iraq; 5 Department of Environmental Studies, Alexandria University, Alexandria, Alexandria Governorate, 163, Egypt

**Keywords:** Orlistat(Xenical™); Anti-obesity drug; High-fat diet; Hepato-nephrotoxicity; Male rats; Metabolic syndrome; Histopathology

## Abstract

**Background:**

HFDs have adverse effects on metabolic health, which puts individuals at risk of obesity and liver and kidney dysfunction. Orlistat is an antihypertensive agent against obesity that prevents the absorption of dietary fats, although its impact on hepatic and renal functions is still disputed. In this study, the dose-dependent effects of orlistat on liver and renal functioning were in male rats on an HFD were assessed.

**Methods:**

There was a control HFD group and three HFD groups with orlistat (360, 480 and 600 mg/kg) on male rats over 60 days. Lipid profiles, liver enzymes (AST, ALT, GGT, ALP), kidney (urea, creatinine, direct and indirect bilirubin), and hematological (hemoglobin, hematocrit, RBC, WBC counts, MCV, MCH, MCHC) serum tests were done. Histopathological analysis of liver and kidney tissues was done.

**Findings:**

Orlistat caused dose-related changes in lipid profiles, increased liver enzymes, and an increased mark of kidney functioning. The hematological parameters were also greatly impaired, and the histopathology indicated structural and tissue damage in both of the organs, more so at higher doses.

**Conclusions:**

Orlistat was used in HFD-conditions in male rats, which resulted in severe dose-related impairment of the liver and kidney. These results highlight the importance of close observation of hepatic and renal functions when using orlistat especially in the high-fat dietary situation.

## 1- Introduction

The increasing obesity epidemic has posed a significant burden on global public health. Obesity and obesity-related diseases including, but not limited to diabetes, hypertension, heart disease, and some cancers, affect millions of people worldwide ( Pan et al., 2023). According to estimates by the World Health Organization, around 1 billion adults globally are obese, and the total cost of obesity-related care will amount to $1.2 trillion by 2030. Given the increasing pace of obesity propagation, health professionals dealing with obesity realize the necessity of fast and efficient interventions ( Rinella et al., 2023). Medication, lifestyle changes, and surgical interference seem to be the most promising strategies in addressing the problem of obesity and related conditions.

Closely associated with the issue of dosage is the concept of toxicity, as overexposure to many other substances can lead to devastating health effects ( Ibadi et al., 2024). Environmental toxins, poor nutrition, and a sedentary lifestyle are just some of the factors that cause obesity and its health consequences. In the midst of the desperate search for solutions, the scientific community identifies Orlistat as an effective remedy that promotes patients’ weight loss, providing people with a chance to conquer obesity ( Dludla et al., 2018). By appreciating this interplay between genetics, environment, and lifestyle, healthcare professionals may be able to develop effective interventions to help stop the obesity epidemic and improve public health.

Over 1.9 billion people were overweight and 650 million were obese in 2023, according to the World Health Organization, suggesting that the global obesity epidemic had reached alarming levels. Obesity is mostly caused by metabolic illnesses such type 2 diabetes, heart disease, non-alcoholic fatty liver disease, and chronic kidney disease. Obesity causes 4.7 million premature deaths annually. In addition to being a trigger for the above aberrations, High-fat diets, a crucial driver of obesity, exacerbate systemic inflammation and oxidative stress. This is followed by lipid accumulation in critical organs such as the liver causing hepatic steatosis, renal lipotoxicity, and multi-organ failure. Although lifestyle interventions remain the foundation of obesity treatment, pharmacological interventions such as the prescription of orlistat are becoming more common due to fears about serious weight-related problems ( Islam et al., 2024).

The pancreatic lipase inhibitor orlistat has a clinically meaningful effect on metabolic indices and weight reduction in obese individuals by reducing dietary fat absorption by 30%. Although the inhibitory effects of orlistat on body weight and serum levels of LDL cholesterol are well established, recent data suggest that orlistat may offer a novel therapeutic option by reducing HFD-induced organ damage, an effect ascribed to its ability to prevent lipid peroxidation and the subsequent inflammatory cascade. However, discordant results have been disclosed regarding hepatorenal toxicity, which suggest that Orlistat may induce systemic toxicity in a dose-dependent manner, thus limiting its application in obese patients with preexisting organ failure based on Available evidence. For example, it has been reported that orlistat-treated rats at supraphysiological doses found equivalent to an intravenous bolus of 4000 mg for humans have higher levels of activated oxidative stress markers. However, it has been recently reported in a clinical safety trial that orlistat has less than 1% bioavailability and scarcely produce systemic toxicity ( Al-Sabah et al., 2023; Huang et al., 2025).

The liver and the kidneys are among the most susceptible to obesity-induced damage. HFD-induced hepatic lipids are encapsulated with NAFLD development, whereas renal lipotoxicity damages glomerular filtration and tubular function. Although recent research indicates that the utilization of orlistat in HFD-fed mice can improve hepatic steatosis and insulin sensitivity through the inactivation of gut microbiota and suppression of de-novo lipogenesis, knowledge of its kidney effects is scarce. This is the first paper to describe the histopathological outcomes of preclinical models. NAFLD and CKD are prevalent comorbidities over 20–30% of obese populations develop and 30–50% demonstrate CKD in the infancy stage. Therefore, learning the organ-specific power of orlistat may help people optimize therapeutic measures ( Ren et al., 2023; Huang et al., 2025).

In male rats given an HFD, this study examines the dose-dependent effects of orlistat on the histology and function of the liver and kidneys. By evaluating biochemical markers and histological changes, we aim to clarify whether orlistat’s benefits in weight management extend to hepatorenal protection or pose unintended risks at therapeutic doses.

## 2- Materials and methods

### 2-1- Preparation of the high-fat diet

Preparation of the high-fat diet was performed according to Altunkaynak (2005) with complete methodological descriptions.

### 2-2- Orlistat drug

Pharmaceutical-grade orlistat was obtained in capsule form (120 mg) from local pharmacies in Diwaniyah, Iraq (Roche, Switzerland). The treatment was administered daily for 60 consecutive days. To ensure dosage precision, the rats were weighed weekly, and the doses (360, 480, and 600 mg/kg) were adjusted accordingly based on their average body weight. The contents of the capsules were freshly dissolved in distilled water before administration via oral gavage.

### 2-3- Experimental animals and design

Twenty-five male Albino rats weighing an average of 190 ± 5 g at two months of age were utilized in this investigation. They came from Al-Qadisiyah University’s College of Science’s Animal House. The Ethics Committee of the College of Applied Sciences at the University of Fallujah (UOF.CAS.06-240519), Iraq, has accepted the study, and all procedures were carried out in compliance with the Committee’s rules and regulations. Prior to the trial, the animals were acclimated for two weeks. The experimental mice had a 12-hour daily photoperiod and were housed in specially designed plastic cages at room temperature (22–25°C). The trial lasted for sixty days. Five equal groups of five rats each were randomly assigned to the animals: the control group, the high-fat diet (HFD) group, and the HFD group that received daily oral gavages of Orlistat (XE) at dosages of 360, 480, and 600 mg/kg for 60 days ( Sabik et al., 2022).

The rats were put to sleep with isoflurane and killed after 60 days. Following their desiccation, blood was drawn from the heart and the Vena Cava and placed in test tubes with heparin, an anticoagulant. One milliliter was extracted for hematological parameters. The plasma was separated from the blood using centrifugation at 860×g for 20 minutes, and it was thereafter kept at -80°C for analysis. Following their separation, the liver and kidney were maintained at -80 °C for further biochemical study after being cleansed with saline. Formalin (10%) is used to preserve portions of specific organs utilized in histological investigations.

Anesthesia of the rat was induced with inhaled isoflurane (3-4 percent of the total volume) with a calibrated vaporizer in an induction chamber and maintained at 1.5-2 percent. In a case where total loss of the pedal reflex had been achieved, euthanasia was done by exsanguine through cardiac puncture, according to AVMA Guidelines of Euthanasia of Animals (2020).

### 2-4- Hematological analysis

The whole blood samples were analyzed for hemoglobin (Hb), blood hematocrit (Ht), red blood cells (RBC), and white blood cells (WBC) shortly after they were obtained. A commercial kit (Diamond Diagnostics, Cat. No. HB102010) was used to measure the concentration of hemoglobin in whole blood. A light microscope with a 400x magnification was used to count red blood cells on a bright-line hemocytometer. Before being counted, blood samples were diluted 200 times with physiological saline (0.9% sodium chloride solution). Ht was tested using micro hematocrit tubes and a hematocrit centrifuge (5 minutes at 16,500 Xg). After blood samples were diluted 20 times with a diluting solution (1% acetic acid and a little amount of Leshman’s stain), WBCs were counted on an American Optics bright line hemocytometer using a light microscope at 100x magnification. Wintrobe indices were calculated in compliance with Wintrobe (2009). Mean corpuscular hemoglobin (MCH) (pg) = Hb (g per 100ml)/ No. of RBCs (per 100 ml), mean corpuscular volume (MCV) (cu μ) = Ht (%)/No. of RBCs (per 100 ml), and mean corpuscular hemoglobin concentration (MCHC) (%) = [Hb (g per 100ml)/Ht (%)] x 100.

### 2-5- Biochemical parameters

According to the method of Armstrong & Carr (1963), the concentration of plasma total protein (TP) was measured using a commercial kit (Diamond Diagnostics, Cat. No. 1001291). The concentration of triglycerides (TG) was determined using a commercial kit (Diamond Diagnostics, Cat. No. 1001311) using the method of Bucolo & David (1973). The method of Richmond (1973) was used to measure total cholesterol and high-density lipoprotein cholesterol (HDL-c) was measured using a commercial kit (Diamond Diagnostics, Cat. No. 1001164). The assay of Assmann (1984) was used to measure low-density lipoprotein cholesterol (LDL-c) using a commercial kit (Diamond Diagnostics, Cat. No. 1001161). The method of Patton & Crouch (1977) was used to measure urea concentration using a commercial kit (Diamond Diagnostics, Cat. No. 1001329) was calculated. Creatinine concentration was measured according to the method Schirmeister et al. (1964) using a commercial kit (Diamond Diagnostics, Cat. No. 1001111). The method of Price et al. (1976) was used to measure both direct and indirect bilirubin concentrations using a commercial kit (Diamond Diagnostics, Cat. No. 1001044).

### 2-6- Enzyme activity

The method of Reitman & Frankel (1957) was used to measure the activity of AST using a commercial kit (Diamond Diagnostics, Cat. No. 260 001) and the activity of ALT (Diamond Diagnostics, Cat. No. 272 001). The method of Persijin & van der Slike (1982) was used to determine γ-GT using a commercial kit (Diamond Diagnostics, Cat. No: 41290). Belfield & Goldberg (1971) technique was used to measure the activity of AlP using a commercial kit (Diamond Diagnostics, Cat. No: 41240). All procedures were conducted strictly according to the manufacturer’s instructions and were performed using commercially available diagnostic kits manufactured by Spectrum Diagnostics.

### 2-7- Histology studies

Liver and kidney tissue samples were embedded in paraffin wax, fixed in a 10% formaldehyde solution, and cut into 5μ μm-thick slices using a microtome. Hematoxylin and Eosin (H&E) stains were applied to the sections, and their morphology was assessed under a microscope ( Durry et al., 1980).

### 2-8- Statistical analysis

The data entered into the computer was examined using IBM SPSS software version 20.0. (IBM Corp., Armonk, NY). The distribution’s normality was confirmed using the Shapiro-Wilk test. The mean and standard error were used to characterize quantitative data. The results’ significance was assessed at the 5% level. The F-test (ANOVA) for normally distributed quantitative variables and the Post Hoc test (Tukey) for pairwise comparisons are utilized when comparing more than two groups.

### 2-9- Ethics approval

This study involved all the animals procedures that were conducted following the formal approval by the Ethics Committee of the College of Applied Sciences of the University of Fallujah, Iraq (Approval No. UOF.CAS.06-240519). All of the experimental procedures, such as handling, housing, anesthesia, and euthanasia, were performed according to the institutional guidelines and the American Veterinary Medical Association (AVMA) Guidelines on the Euthanasia of Animals (2020).

## 3- Results and discussion

The hematological indicators of male rats administered Orlistat (XE) at dosages of 360, 480, and 600 mg/kg and fed a high-fat diet (HFD) are summarized in Tables 1 and 2. These indicators include hemoglobin (Hb), hematocrit (Ht), red blood cell count (RBC), white blood cell count (WBC), mean corpuscular volume (MCV), mean corpuscular hemoglobin (MCH), and mean corpuscular hemoglobin concentration (MCHC). Hematological indicators did not alter statistically significantly (P > 0.05) between the HFD group and the control group (regular diet). Within the context of the current study, the value of P above 0.05 means that the observed biochemical difference between groups was not statistically significant based on the applied tests, but does not rule out the existence of biological or histopathological changes. Actually certain doses of orlistat demonstrated some tissue alterations in the kidneys and liver even though there were no statistically significant biochemical changes. Thus, the P-values interpretation was supplemented by the histological evidence, which is more exhaustive and biologically significant to evaluate the impairment of organs.

**Table 1.** Effects of Orlistat (XE) at doses of 360, 480, 600 mg/kg on hemoglobin (Hb; g/dL), hematocrit (Ht; (%), red blood cell count (RBC; x106/ml), and white blood cell count (WBC; x10 ^3^/ml) of male rats fed a high-fat diet (HFD).

Experimental groups	Parameter			
	Hb	Ht	RBCs	WBCs
**Control**	11.7 ^b^ ± 0.42	33.9 ^ab^ ± 1.41	6.22 ^b^ ± 0.22	13.0 ^a^ ± 1.01
**HFD**	12.3 ^ab^ ± 0.05	34.9 ^ab^ ± 0.04	6.42 ^ab^ ± 0.02	12.6 ^a^ ± 0.23
**HFD+XE-360**	11.5 ^b^ ± 0.01	32.3 ^b^ ± 0.02	6.06 ^b^ ± 0.01	10.1 ^a^ ± 1.43
**HFD+XE-480**	12.7 ^a^ ± 0.22	36.2 ^a^ ± 0.72	6.92 ^a^ ± 0.12	11.7 ^a^ ± 2.23
**HFD+XE-600**	12.7 ^ab^ ± 0.11	34.1 ^ab^ ± 0.29	6.33 ^b^ ± 0.08	11.1 ^a^ ± 0.01

Results are expressed as Mean ± SE. ^abc^Mean values within a column not sharing common superscript letters were significantly different, *p *< 0.05.

**Table 2.** Effects of Orlistat (XE) at doses of 360, 480, 600 mg/kg on mean corpuscular volume (MCV; fL), mean corpuscular hemoglobin (MCH; pg), and mean corpuscular hemoglobin concentration (MCHC; g/dL) of male rats fed a high-fat diet (HFD).

Experimental groups	Parameter		
	MCV	MCH	MCHC
**Control**	54.3 ^a^ ± 0.31	18.9 ^a^ ± 0.02	34.7 ^c^ ± 0.23
**HFD**	54.4 ^a^ ± 0.19	19.2 ^a^ ± 0.14	35.2 ^bc^ ± 0.12
**HFD+XE-360**	53.3 ^b^ ± 0.06	19.1 ^a^ ± 0.02	35.8 ^a^ ± 0.02
**HFD+XE-480**	52.2 ^c^ ± 0.15	18.4 ^b^ ± 0.02	35.2 ^bc^ ± 0.10
**XE-600**	53.9 ^ab^ ± 0.25	19.0 ^a^ ± 0.08	35.4 ^ab^ ± 0.02

Results are expressed as Mean ± SE. ^abc^Mean values within a column not sharing common superscript letters were significantly different, *p *< 0.05.

Hematological parameters (Hb, Ht, RBC, WBC, MCV, MCH, and MCHC) did not significantly change between the control, high-fat diet (HFD), and Orlistat -treated groups, indicating that neither the administration of Orlistat (360–480–600 mg/kg) nor the HFD caused hematotoxicity or systemic anemia in male rats under the experimental conditions. These findings align with studies demonstrating that orlistat primarily acts locally in the gastrointestinal tract to inhibit pancreatic lipase, with limited systemic absorption and minimal direct effects on hematopoietic parameters ( Hauptman et al., 2000).

The absence of HFD-induced hematological alterations in the current study stands in contrast with several previous reports of some kinds of obesity-associated anemia of chronic disease, presumed to be caused by chronic inflammation or iron control. However, the short study interval of just 60 days and lack of critical metabolic abnormalities in the HFD group may contribute to this discrepancy. Indeed, research in diet-induced obese mice have revealed that such hematological effects usually become evident only after more extended attention or in the context of co-morbid conditions such as insulin opposition ( Kassebaum et al., 2014).

The unmodified WBC counts in all groups in addition to the above sheds more evidence that Orlistat did not induce systemic inflammation or immunosuppression although well-tolerated gastrointestinal side effects including steatorrhea. This is consistent with clinical reports that orlistat does not raise inflammatory markers such as CRP or IL-6 in obese patients ( Yesilbursa et al., 2005). While, Zahkouk et al. (2019) observed increased W.B.C. and decreased R.B.C., HB, and Hct% in the HFD group. Rahi and Khudhair (2024) found that male rats treated with HFD+ tocotrienol showed differences in RBC and MCH. Additionally, the WBC of male rats given HFD alone was significantly higher than that of the control group. To assess the combined effects of Orlistat and HFD on hematopoiesis, longer-term research is required. Investigating Orlistat impact in comorbid models (e.g., diabetic-obese rats) could reveal context-dependent hematotoxicity.

Interestingly, no significant changes in hematological parameters were found in the current research even with the intake of a high-fat diet and (orlistat). This is contrary to some of the past reports that obesity or large amount of fat in the diet might cause anemia or inflammation. Another reason could be the fact that the experimental range (60 days) is quite short and may not be enough to induce chronic anemia or systemic inflammatory reactions. Also, the rats were not found to have critical metabolic disturbances or extreme obesity, which in many cases is necessary to bring about the effect of hematological changes. In addition, orlistat mainly works in the gastrointestinal tract to prevent fat absorption, and has the least effects of the system on hematopoiesis, which is agreeable with the fact that the RBC, WBC, Hb, and Ht levels remained unchanged in this study.

Table 3 shows the total protein (TP) and lipid profile parameters, such as triglycerides (TG), high-density lipoprotein cholesterol (HDL-c), and low-density lipoprotein cholesterol (LDL-c), in male rats fed a high-fat diet (HFD) and given Orlistat (XE) at dosages of 360, 480, and 600 mg/kg. Between the HFD group and the control group (normal diet), there was no statistically significant difference in TP levels (P > 0.05). The three Orlistat -treated groups and the control and HFD groups did not vary significantly (P > 0.05) in TP. However, TG, HDL-c, and LDL-c values in all Orlistat -treated groups (360, 480, and 600 mg/kg) revealed substantial improvements (P < 0.05) in comparison to the control and HFD groups.

**Table 3.** Effects of Orlistat (XE) at doses of 360, 480, 600 mg/kg on total protein (TP; g/dL), triglyceride (TG; mg/dL), high-density lipoprotein-cholesterol (HDL-c; mg/dL), and low-density lipoprotein-cholesterol (LDL-c; mg/dL) of male rats fed a high-fat diet (HFD).

Experimental groups	Parameter			
	TP	TG	HDL-c	LDL-c
**Control**	6.60 ^a^ ± 0.08	52.60 ^bc^ ± 1.32	15.55 ^b^ ± 0.68	45.5 ^a^ ± 2.90
**HFD**	6.80 ^a^ ± 0.12	49.60 ^c^ ± 6.01	16.0 ^b^ ± 0.45	29.1 ^c^ ± 0.81
**HFD+XE-360**	6.75 ^a^ ± 0.02	66.25 ^ab^ ± 3.54	22.60 ^a^ ± 1.47	33.8 ^b^ ± 1.15
**HFD+XE-480**	6.94 ^a^ ± 0.03	72.85 ^a^ ± 2.89	18.60 ^ab^ ± 2.36	29.4 ^bc^ ± 3.74
**XE-600**	6.64 ^a^ ± 0.22	54.45 ^bc^ ± 0.02	15.50 ^b^ ± 0.93	29.3 ^bc^ ± 2.63

Results are expressed as Mean ± SE. ^abc^Mean values within a column not sharing common superscript letters were significantly different, *p *< 0.05.

The present study demonstrated that total protein (TP) levels remained unchanged across control, high-fat diet (HFD), and Orlistat (XE)-treated groups, indicating that neither the HFD nor the administration of Orlistat at doses of 360, 480, and 600 mg/kg significantly impacted the overall protein metabolism or hepatic synthetic function in male rats. This finding aligns with previous research showing that orlistat primarily targets fat absorption without adversely affecting protein metabolism or liver synthetic capacity in animal models and humans ( Padwal & Majumdar, 2007; Hauptman et al., 2000).

However, in all Orlistat -treated groups, the lipid profile parameters TG, HDL-c, LDL-c were significantly altered compared to both the control and HFD groups. More precisely, treatment with orlistat resulted in a significant decrease in TG and LDL-c in addition to an increase in levels of HDL-c. These outcomes can be explained by the described pharmacological factor of orlistat: it is a pancreatic lipase inhibitor that prevents the breakdown of fat and thus promotes a better lipid profile caused by obesity or an HFD ( Torgerson et al., 2004; Gao et al., 2024). These lipid-modulating effects have been further validated by clinical and preclinical studies. Specifically, Torgerson et al. (2004) discovered that orlistat improved cardiovascular risk profiles by lowering blood TG and LDL-c levels and raising HDL-c levels in patients who were noticeably obese. However, these findings differ from those of Annamalai et al. (2016) that the HFD-induced rats were prompted to raise triacylglycerol, LDL, and lower high-density lipoprotein. High-fat diet and the Orlistat showed significant differences in the levels of cholesterol, triacylglycerol, and LDL, as well as noticeable differences in HDL compared to high-fat diet, according to the results, which also showed a significant departure at the level of triacylglycerol and LDL compared to the control group and a significant difference at the level of HDL ( Fahmi, 2021).

Since total protein was unchanged although there were significant improvements in the lipid profile, it can be argued that the lipid-lowering effects of orlistat are not associated with adverse effects on protein metabolism and liver function. This can be explained by the existing report stating that orlistat treatment did not result in deterioration of serum albumin and total protein in obese patients ( Hauptman et al., 2000).

The correlation between lipid metabolism and organ functioning offers the additional interpretation of the biochemical and histological results of the current study. Dyslipidemia caused by high-fat diet facilitates overexpression of lipid influx to the liver and kidney, which is part of hepatic steatosis, oxidative stress and renal lipotoxicity. Interference with this gut liver kidney axis is viewed as a key route by which dietary fat excess causes progressive hepatorenal damage. Orlistat only partially regulates this axis by stopping the intestinal pancreatic lipase, which prevents absorption of dietary fats, and restricts the systemic delivery of triglycerides and free fatty acids. Subsequently, reduced lipid supply reduces hepatic fat deposition, diminishes renal fatty infiltration and inhibits oxidative and inflammatory signaling in these interdependent organs. Such a mechanistic interaction is the reason behind the enhanced lipid profile and the partial restoration of liver and kidney histology in the orlistat-treated groups, especially at the 360 and 480 mg/kg dosage. Nevertheless, the fact that structural changes still persist with increased doses is a hint that lipid metabolism modulation should not be given so much consideration to fully offset chronic HFD load, highlighting the complexity of the gutliverkidney axis in organ dysfunction related to obesity.

Table 4 summarizes the serum urea, creatinine, direct bilirubin, and indirect bilirubin of male rats fed a high-fat diet (HFD) and given Orlistat (XE) at dosages of 360, 480, and 600 mg/kg. There were significant changes ( *P* < 0.05) observed in the tested parameter between the control group (normal diet) compared with the HFD and the three Orlistat -treated groups (360, 480, and 600 mg/kg).

**Table 4.** Effects of Orlistat (XE) at doses of 360, 480, 600 mg/kg on serum urea (mg/dL), creatinine (mg/dL), direct bilirubin (mg/dL), and indirect bilirubin (mg/dL) of male rats fed a high-fat diet (HFD).

Experimental groups	Parameter			
	Urea	Creatinine	Direct Bilirubin	Indirect Bilirubin
**Control**	43.10 ^ab^ ± 0.04	0.56 ^a^ ± 0.03	0.20 ^ab^ ± 0.0	0.40 ^b^ ± 0.08
**HFD**	42.05 ^abc^ ± 3.0	0.44 ^b^ ± 0.01	0.35 ^a^ ± 0.02	0.45 ^b^ ± 0.06
**HFD+XE-360**	39.80 ^bc^ ± 0.81	0.47 ^b^ ± 0.02	0.35 ^a^ ± 0.10	0.53 ^ab^ ± 0.05
**HFD+XE-480**	48.05 ^a^ ± 0.44	0.44 ^b^ ± 0.002	0.25 ^ab^ ± 0.02	0.62 ^ab^ ± 0.01
**XE-600**	36.29 ^c^ ± 1.51	0.57 ^a^ ± 0.01	0.13 ^b^ ± 0.01	0.68 ^a^ ± 0.05

Results are expressed as Mean ± SE. ^abc^Mean values within a column not sharing common superscript letters were significantly different, *p *< 0.05.

HFD affects the kidney by increasing urea and creatinine ( Amin et al., 2014). According to Fahmi’s (2021) findings, the high-fat diet significantly raised the levels of urea and creatinine when compared to the control group; however, there were no significant changes between the high-fat diet and Orlistat.

Additionally, the documented increase in blood urea and creatinine in male rats after high-fat meals were administered is in line with research on the connection between high-fat diets and renal failure. KF Diet-induced oxidative stress and lipid deposition in the renal tissues lead to inflammation and dysfunction of the glomerular filtration and tubules, increasing isaac, and markers of kidney injury ( Muller et al., 2019; Prem and Kurian, 2021).

For example, Muller et al. (2019) established that HFD feeding of the murine animal model leads to glomerular retraction, lower GFR, and increases glucose synthesis, and pro-inflammatory cytokines including IL-6, consistent with reparation and inflammatory mechanisms involved in the pathogenesis of renal impairment. ( Prem and Kurian, 2021) demonstrated that 16 weeks of HFD significantly exhausted eGFR and increased plasma creatinine in treated groups, reinforcing the nephrotoxic consequences of lipid toxicity duration.

The non-significant differences in urea and creatinine levels between the HFD and Orlistat -treated groups in our investigation indicate that Orlistat therapy at dosages of 360, 480, and 600 mg/kg did not exacerbate HFD-induced renal impairment. However, and in contrast with previous works, Tousson et al. (2018): clearly showed that orlistat caused a significant rise in serum creatinine and urea in normal rats supporting the expected nephrotoxic effect at usual medication doses. However, the lack of toxicity observed in our HFD model could be attributed to adapting compensatory mechanisms in the overweight rat or differences in the study model as regard treatment duration. Importantly, the most recognized orlistat mechanism of action is directly combating dietary fat absorption, which directs lipid-induced renal lipotoxicity, to indirectly or subsequently un-enhances HFD-induced oxidative stress and inflammation ( Ahmed et al., 2024).

Further research is warranted on the large changes in direct and indirect bilirubin observed in Orlistat -treated groups. It is possible that orlistat impacts bilirubin dynamic because the drug interferes with lipid metabolism, which employs both hepatic and renal excretory pathways. In one study, Nishioka et al. (2003) orlistat increased the fecal excretion of unconjugated bilirubin found in Gunn rats, presumably by reducing the systemic availability of bilirubin. While our findings demonstrate a mediating effect of Orlistat on bilirubin homeostasis, the precise mechanism is unknown, including changes in hepatic conjugation or renal excretion, and should be further investigated.

The hepatorenal changes which were described in the current study can be attributed to a number of mechanistic mechanisms which have been linked to the exposure to orlistat in the past. It is shown that orlistat may cause oxidative stress caused by overproduction of reactive oxygen species (ROS), which results in lipid peroxidation and degradation of antioxidant defense mechanisms SOD, CAT, and GSH. This oxidative imbalance can be one of the factors that led to the increase of liver enzymes and structural hepatic degeneration that was observed in the treated groups. Also, orlistat is said to cause inflammatory reactions by upregulating pro-inflammatory cytokines (e.g., TNF- a, IL- 6), which add to the aggravation of hepatocellular and renal damages. In extreme situations, there is also a chance of oxidative and inflammatory stress during long durations, leading to the activation of apoptosis-related pathways, which cause cellular necrosis and disruption of tissues. Even though our study has not directly measured the molecular markers, the biochemical and histopathological results on the various doses are in agreement with the previously detailed mechanisms, which is why it is possible to conclude that orlistat causes dose-dependent hepatorenal toxicity under the conditions of HFD.

Alkaline phosphatase (AIP), gamma-glutamyl transpeptidase test (GGT), alanine aminotransferase (ALT), and aspartate aminotransferase (AST) activity in the blood of male rats given 360, 480, and 600 mg/kg of Orlistat and fed a high-fat diet (HFD). According to Table 5, the liver enzyme activity of the HFD group was considerably lower than that of the control group that was fed a regular diet. These indices showed a substantial reduction (P < 0.05) in the three Orlistat -treated groups (360, 480, and 600 mg/kg) as compared to the control and HFD groups.

**Table 5.** Effects of Orlistat (XE) at doses of 360, 480, 600 mg/kg on serum enzyme activities (AST, ALT, GGT, and AIP) of male rats fed a high-fat diet (HFD).

Experimental groups	Parameter			
	AST (IU/L)	ALT (IU/L)	GGT (IU/L)	AIP (IU/L)
**Control**	302 ^b^ ± 49.3	99 ^a^ ± 27.5	12.6 ^b^ ± 0.21	298 ^ab^ ± 27.0
**HFD**	176 ^b^ ± 4.8	61 ^ab^ ± 11.3	14.7 ^a^ ± 0.56	209 ^a^ ± 5.9
**HFD+XE-360**	85 ^a^ ± 2.6	29 ^b^ ± 0.5	10.1 ^c^ ± 0.04	349 ^a^ ± 25.18
**HFD+XE-480**	141 ^b^ ± 11.1	30 ^b^ ± 2.5	13.7 ^ab^ ± 0.52	378 ^a^ ± 22.2
**XE-600**	117 ^b^ ± 29.3	27 ^b^ ± 0.6	12.7 ^b^ ± 0.62	334 ^a^ ± 34.1

Results are expressed as Mean ± SE. ^abc^Mean values within a column not sharing common superscript letters were significantly different, *p *< 0.05.

Aspartate aminotransferase (AST; IU/L), Alanine aminotransferase (ALT; IU/L), Gamma-Glutamyl Transpeptidase (GGT; IU/L), and Alkaline Phosphatase (AIP; IU/L).

When compared to the control group, the high-fat diet and Orlistat groups showed substantially lower liver enzyme activity of AST, ALT, GGT, and ALP, indicating that Orlistat is efficient in lowering hepatic stress in male rats. These results went against those seen in the usual high-fat-diet model, where liver enzyme levels were high and associated with hepatic damage, characterized by fatty liver and inflammation ( Svegliati-Baroni et al., 2006). However, the observed decrease in enzyme activities aligns with studies demonstrating that orlistat mitigates liver damage by reducing lipid accumulation and oxidative stress in obesity-related models ( Zakaria et al., 2021; Curran and Scott, 2004).

Although HFD is often related to hepatic steatosis and increased LFTs, the decrease in AST, ALT, GGT, and ALP in the present study could be a result of adaptive metabolic reactions or model distinctions, including feed composition and duration. The short-term exposure to HFD may not have caused severe hepatocellular destruction to increase the levels of these enzymes significantly ( de Freitas Carvalho et al., 2019).

Orlistat’s inhibition of dietary fat absorption reduces lipid infiltration into the liver, thereby alleviating metabolic strain on hepatocytes. This mechanism is supported by studies showing that orlistat lowers liver enzyme levels in rodent models of obesity by improving lipid metabolism and reducing oxidative stress ( Curran and Scott, 2004; Zakaria et al., 2021). For example, Curran and Scott (2004) found that orlistat significantly reduced ALT and AST in obese people with non-alcoholic fatty liver disease (NAFLD), which was associated with lower hepatic fat content. According to Hamza and Alsolami’s (2023) findings, the HFD male rat group had higher levels of liver enzymes (AST and ALT), however orlistat significantly reduced these levels and brought them back to nearly normal levels.

**Figure 1.**
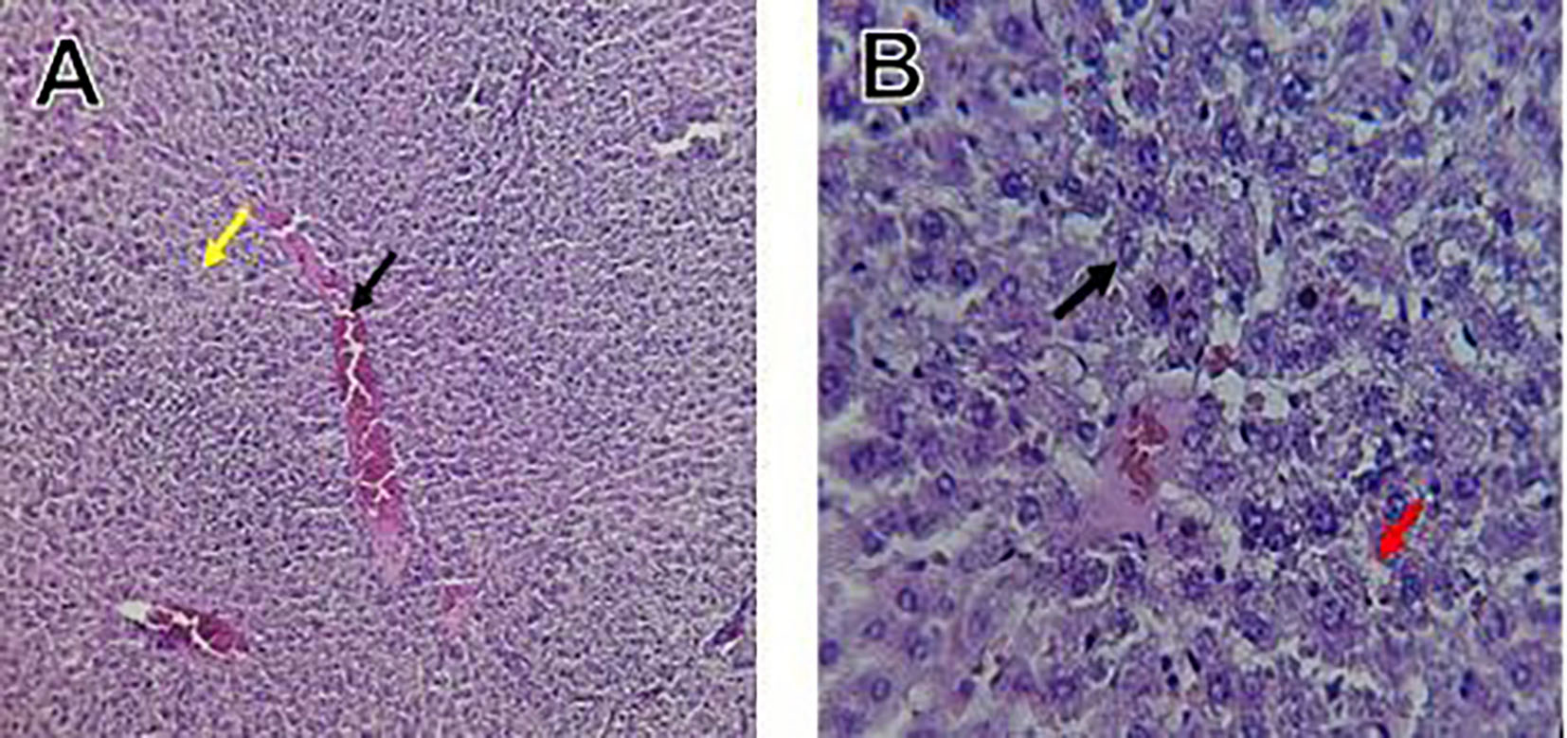
The control group shows normal hepatocyte architecture, central vein (C.V.), normal blood sinusoids (S), and hepatocytes (black arrows).

**Figure 2.**
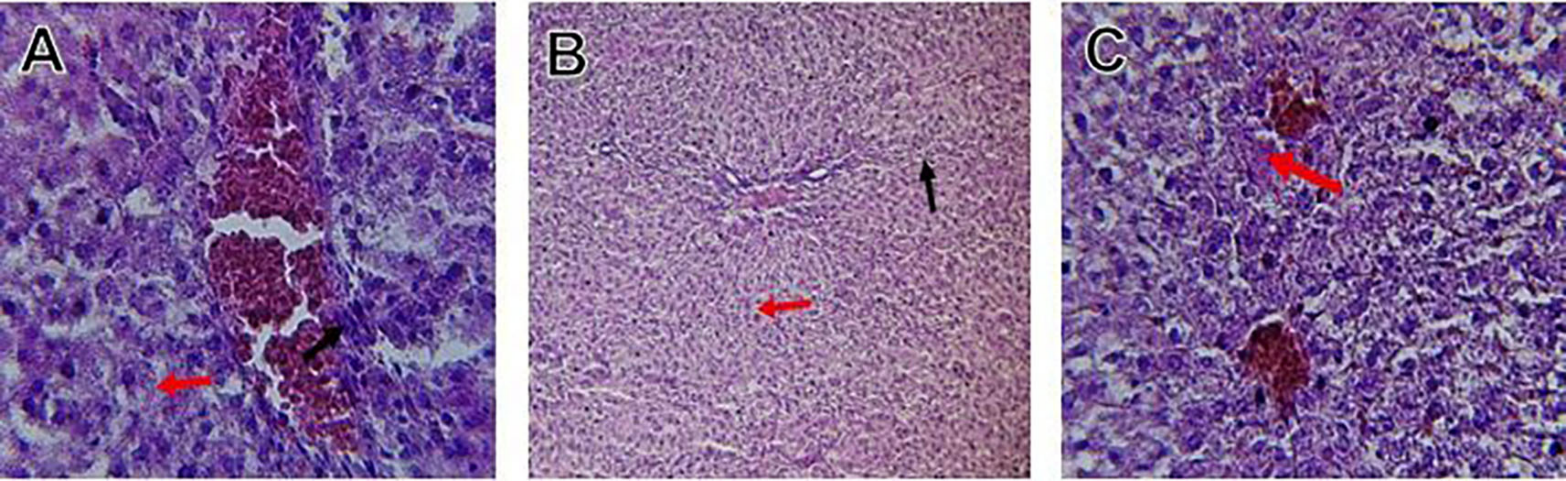
Rats fed a high-fat diet (HFD) group show Lobular infiltrate by chronic inflammatory cells (**), with hepatocytes disarray and cloudy swelling be liver hepatocytes (sinuosoid), apoptotic cells with curvy arrow.

**Figure 3.**
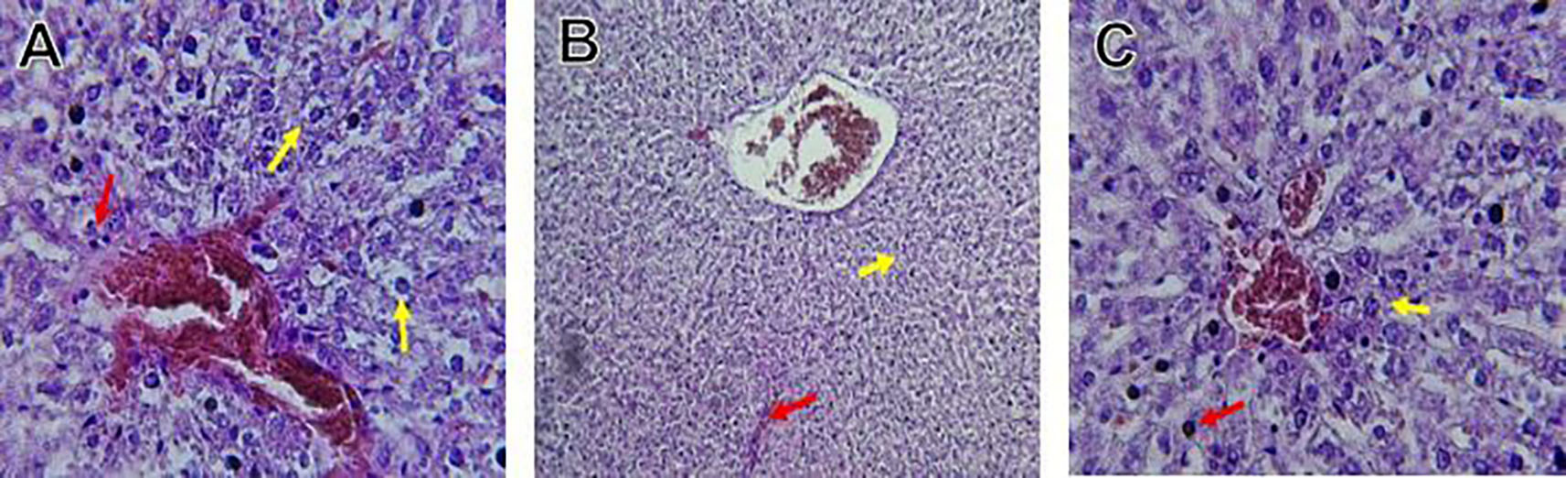
Rats fed a high-fat diet (HFD) and treated with Orlistat (XE-360) shows a slight lobular infiltrate by chronic inflammatory cells, with hepatocytes disarray and cloudy swelling be liver epatocytes (sinuosoid), apoptotic cells.

**Figure 4.**
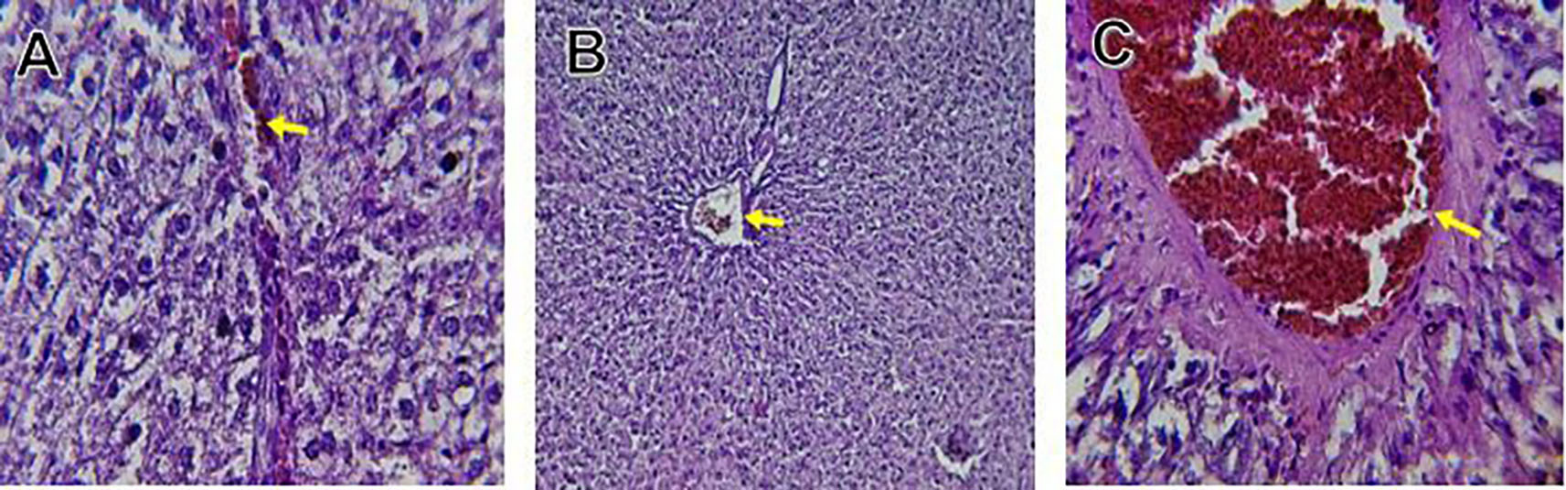
Rats fed a high-fat diet (HFD) and treated with Orlistat (XE-360) show marked dilatation, congestion in the portal vein.

**Figure 5.**
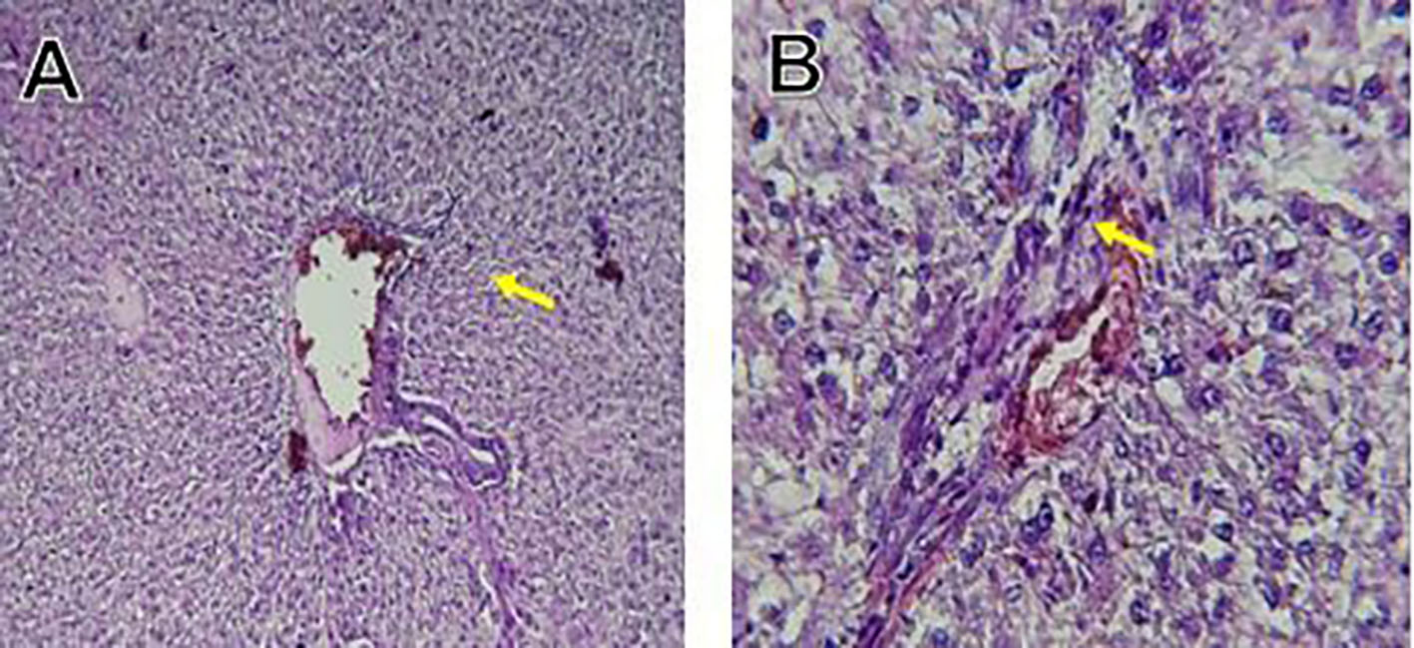
Rats fed a high-fat diet (HFD) and Orlistat (XE-600) showed histological changes induced were slightly reduced, with slight inflammatory cells.

Histological slices of the liver from the control group demonstrate normal blood sinusoids, hepatocytes, central veins, and hepatocyte architecture. HFD-inflicted a severe liver cell injury, and it is evident from the liver histological changes, which include; sinusoidal space, liver sinusoid, toxic drugs, and apoptotic cells are in Figure 1. High-fat diet group HFD, Lobular infiltrate by chronic inflammatory cells, hepatocytes disarray, and cloudy swelling be liver epatocytes; sinuosoid, apoptotic cells with curvy arrow are in Figure 2. HFD treatment with Orlistat; XE-360, slight lobular infiltrate by chronic inflammatory cells, hepatocytes disarray and cloudy swelling be liver epatocytes; sinuosoid, apoptotic cells are in Figure 3. HFD treatment with Orlistat; XE-360, marked dilatation; congestion portal vein are in Figure 4. HFD treatment with Xeni Orlistat cal; XE-600, histological changes induced were slightly reduced, slight inflammatory cells are in Figure 5.

The histological findings of this study reveals the hepatoprotective effects of Orlistat on high-fat fed male rats against the liver damage. Liver slices from the control group had typical hepatic architecture with intact hepatocytes, central veins, and blood sinusoids, as seen in Figure 1. Figure 2 illustrates the significant pathological abnormalities that the HFD group had, including lobular trafficking of inflammatory infiltrates, hepatocyte clutters, hazy edema, and apoptotic bodies. These results are consistent with the finding of Hamza and Alsolami (2023) that HFD caused liver hypertrophy, eosinophilia, and necrotic foci to formation. Zakaria et al. (2021) had earlier reported degenerated hepatocytes with steatosis and portal inflammation on a 16-week HFD fed model.

Orlistat treatment at 360 mg/kg partially attenuated these changes, with a reduction in lobular inflammation and cloudy swelling ( Figure 3); however, residual portal vein congestion persisted ( Figure 4). The areas showed further reduction at the higher dose (XE-600, Figure 5) which implies a dose-dependent therapeutic effect. This is in line with Fahmi (2021) who reported that Orlistat prevents HFD-induced fatty degeneration and inflammatory infiltration while increasing the expansion of hepatic sinusoids in the HFD-fed rats. The incomplete normalization of the liver architecture using even higher doses might be due to the residual accumulation of lipid or irreversible fibrosis of prolonged HFD exposure, similar to the NASLD models ( Zakaria et al., 2021).

Orlistat’s hepatoprotective effect is probably due to its inhibition of pancreatic lipase, which results in diminished fat absorption by the intestine and thus decreased hepatic lipids ( Zakaria et al., 2021). Orlistat reduces steatosis, oxidative stress, and inflammation by diminishing lipid influx, among the primary sources of HFD-induced liver injury. Hamza and Alsolami (2023) confirmed the previous mechanism, noting that orlistat reduced lipid overloading and related necroinflammation, and meanwhile returned the hepatocyte to normal architecture.

**Figure 6.**
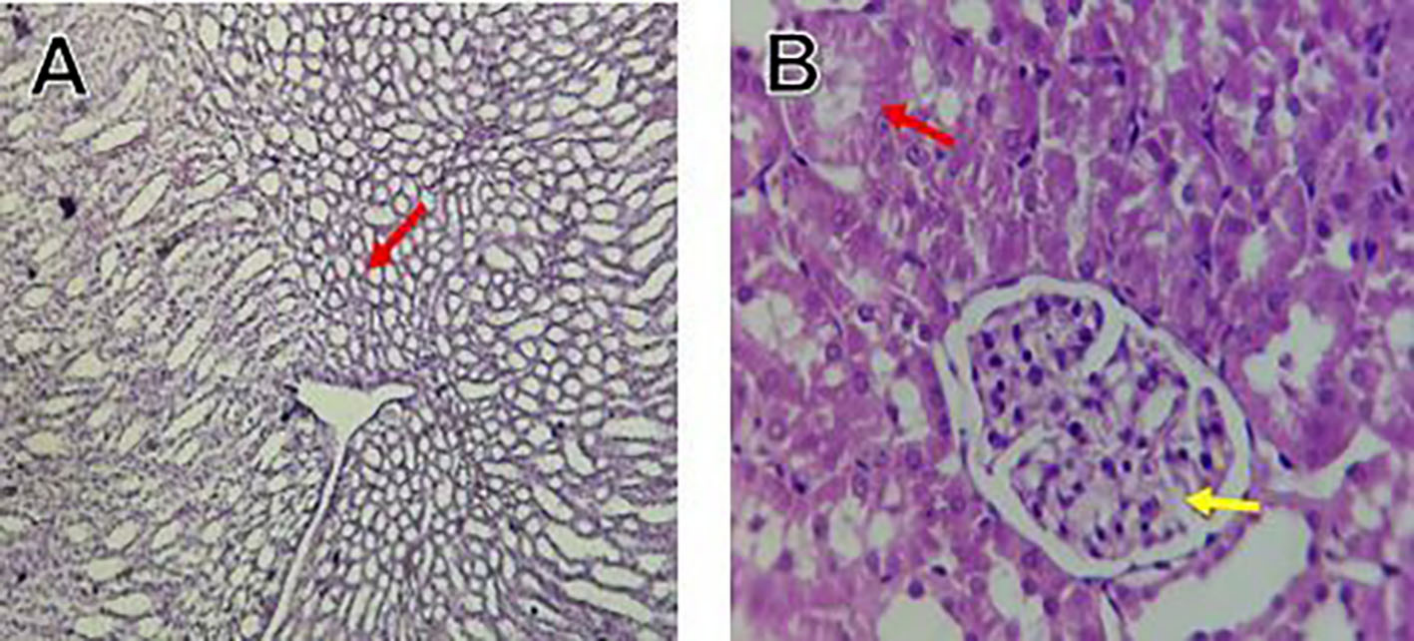
The control group shows a normal histological structure of a kidney. The renal tubules and glomeruli appear healthy and well-defined, indicating no apparent pathological changes.

**Figure 7.**
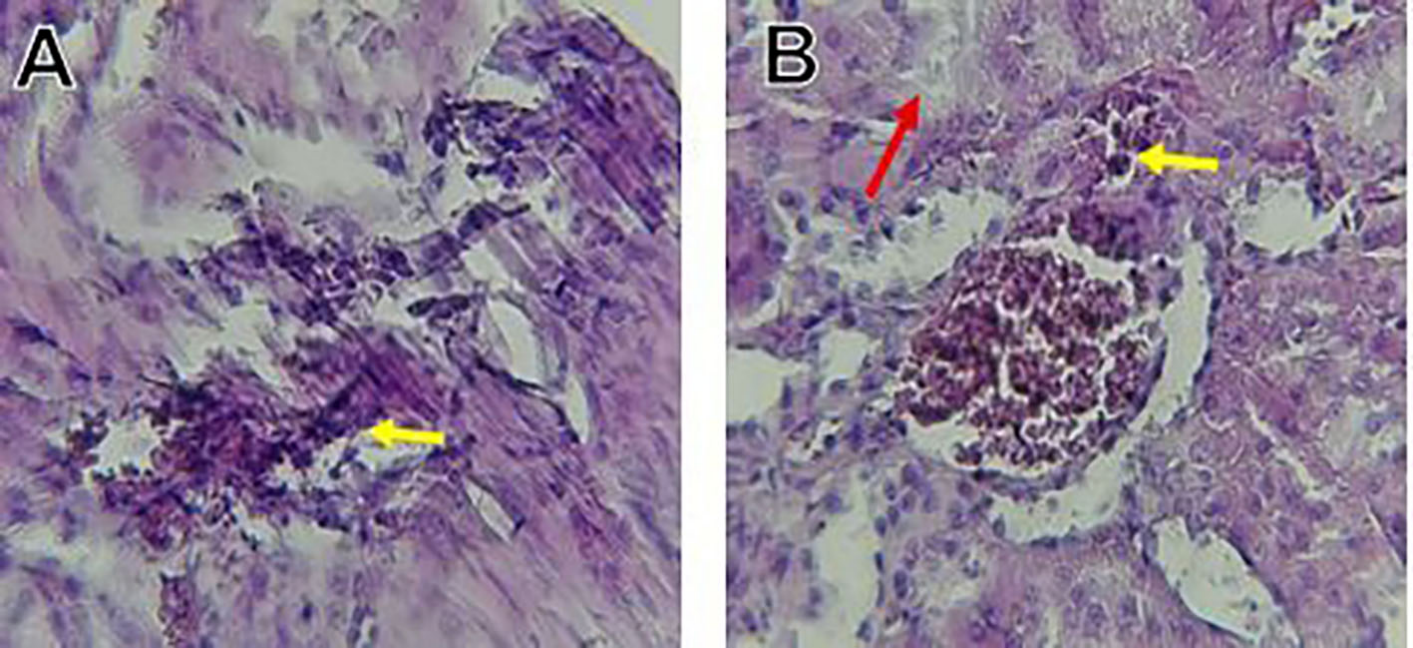
Rats fed a high-fat diet (HFD) group show an accumulation of lipid droplets within the renal tubules, indicating fat deposition and potential cellular damage.

**Figure 8.**
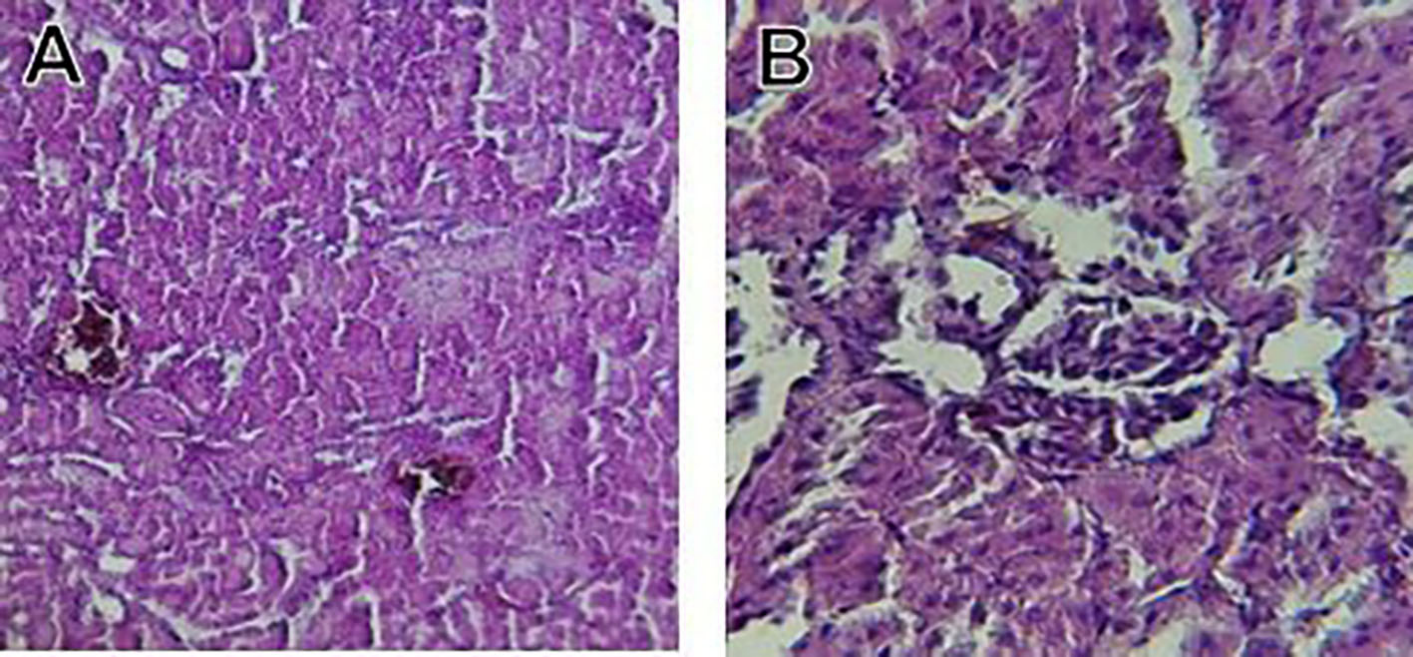
Rats fed a high-fat diet (HFD) and treated with Orlistat (XE-360) appear relatively normal, with no significant pathological changes observed, suggesting that Orlistat at this dose might have a protective effect against the fat-induced damage.

**Figure 9.**
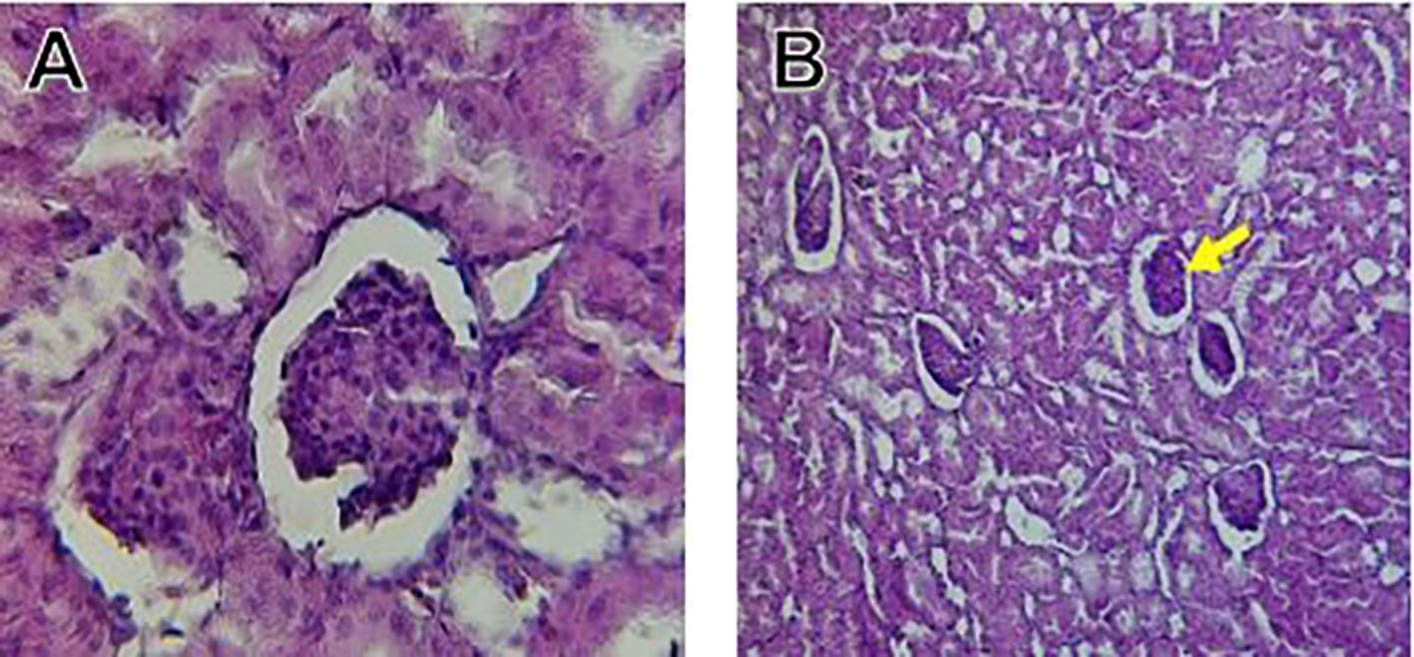
Rats fed a high-fat diet (HFD) and treated with Orlistat (XE-480) appear mostly normal, although there might be some slight changes in the structure of the renal tubules, suggesting a possible mild effect of the high-fat diet that is being mitigated by the dose of XE.

**Figure 10.**
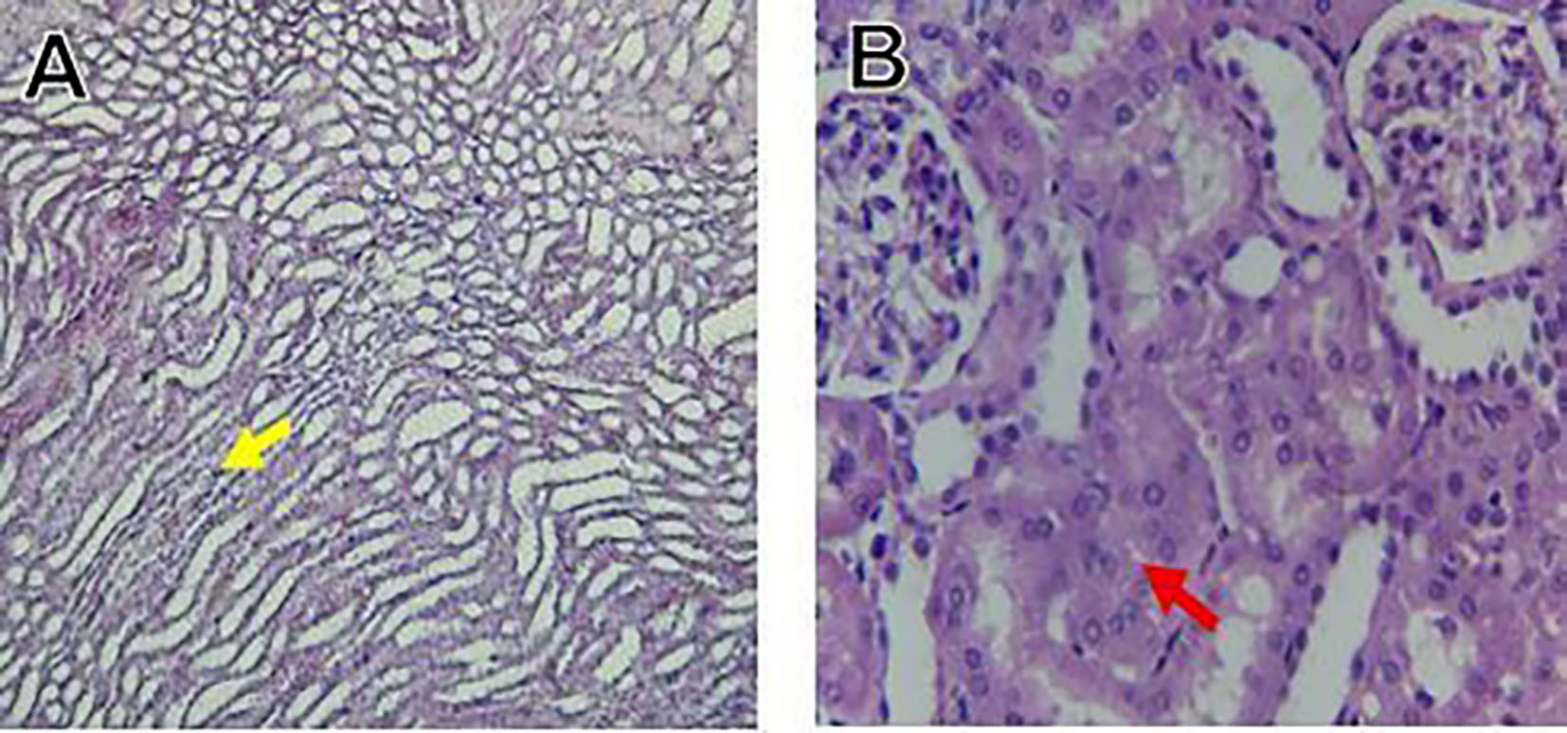
Rats fed a high-fat diet (HFD) and treated with Orlistat (XE-600) appear mostly normal, although there might be some slight changes in the structure of the renal tubules, suggesting a possible mild effect of the high-fat diet that is being mitigated by the dose of XE.

The kidney histological sections of the control, HFA, and HFD groups treated with XE at 360, 480, and 600 mg/kg are displayed in Figures 6– 10. The kidney segment has a normal morphology, as seen in Figure 6 for the control group. Renal tubules are well-studied and functional, and the glomeruli are also normal, which mean there is no apparent pathology. Regarding the HFD group in Figure 7, there are lipid droplets in the renal tubules that can indicate fat deposition and possible damage to cells. Figure 8, which represents HFD treated XE-360, looks relatively normal, and no distinct pathology can be seen. This means that Orlistat at such a dose may have some kind of protective effect against fat-induced damage. On the slide for HFD treated XE-480, shown in Figure 9, the kidney sections are mostly normal, however, the structure of the renal tubule, which is slightly different indicates that there may be some mild effect of HFD, which is “reversed” by the XE dose. The slide for HFD treated with XE-600 is present at Figure 10.

The kidney histology of HFD rats revealed severe pathological modifications, and these features include lipid droplet build up in renal tubules, glomeruluar atophy, necrosis, and tubular dissociation. These outcomes aligned with Salim et al. (2018), whereas HFD-induced renal injury models promoted glomerulosclerosis, segmental necrosis, tubula defects, and mononuclear cell infiltration. Lipid accumulation in renal tissue is critical for lipotoxicity, inflammation, and progressive nephron impairment in kidney illness related to obesity ( Zakaria et al., 2021).

Somewhat amelioration of the renal changes following Orlistat treatment at 360, 480, and 600 mg/kg appears to have occurred, as revealed by histological sections consistent with nearly normal glomeruli and tubules, and diminished fatty infiltration. Likewise, Fahmi (2021) revealed a partial restoration of the renal architecture and improvement of fatty degeneration after orlistat treatment in HFD-induced rats. This amelioration might have been ascribed to the lower body’s lipid load and the amelioration of oxidative stress and inflammation, as reported by Zakaria et al. (2021) and Ren et al. (2023).

Our results show that there is a definite dose-related effect of orlistat on the hepatic and renal functions. This is because the significant degenerative changes were observed at 480 and 600 mg/kg which is accompanied by minor histological changes which are an indication of potential protective effect at 360 mg/kg. This dose reward relationship shows emphasis on dose selection in order to reduce hepatic and renal toxicity, as in Table 6.

**Table 6.** Orlistat effect on the liver and kidneys at various strephenyl doses.

Dose (mg/kg)	Liver function	Kidney function	Histopathological changes (Liver)	Histopathological changes (Kidney)
360	ALT, AST: slight increase (within normal range)	Creatinine, BUN: slight increase	Mild hepatocyte vacuolation; minimal inflammatory infiltration	Mild tubular dilation; intact glomeruli
480	ALT, AST: moderate increase	Creatinine, BUN: moderate increase	Moderate hepatocyte degeneration; scattered necrosis; mild inflammation	Moderate tubular degeneration; mild glomerular changes
600	ALT, AST: significant increase	Creatinine, BUN: significant increase	Severe hepatocyte necrosis; marked inflammation; early fibrosis	Pronounced tubular necrosis; glomerular degeneration; interstitial inflammation

The current results suggest that orlistat has a biphasic pattern of activity with lesser doses having mild protective or neutral effect on the organism whereas greater doses (480-600 mg/kg) have overt hepatorenal toxicity. According to the measured dose-dependent histopathological and biochemical changes, the findings indicate that the therapeutic margin within which orlistat is safe is small. This is in line with past studies that indicate that excessive suppression of fat intake can overstretch the gut-liver axis resulting in the build-up of lipids, inflammatory response and metabolic strain.

Even though current doses applied to the animal model are scaled with animal weight, its findings highlight the possible risk of this high dose or long-term exposure to orlistat in humans. Persons with pre-existing liver or kidney diseases can be especially susceptible because any moderate change in lipid metabolism or oxidative homeostasis would worsen already existing organ damage. Thus, orlistat in lower dose can be effective as short-term therapeutic treatment and needs caution but higher dosage or longer administration but clinical follow-up of liver and kidney indicators is highly encouraged. These observations highlight the need to conduct more translational research to establish a more precise safety margin as well as to identify the clinical meaning of dose-related hepatorenal changes in the animal model.

## 4. Conclusion and future work

Summarized, the current work demonstrates that prolonged exposure of the male rats fed HFD to Orlistat at doses from 360 to 600 mg/kg exacerbates the pre-existing hepatic and renal dysfunction. On the one hand, the maximal daily dose was used to simulate the intended clinical action of Orlistat to prevent fat absorption and cause mild side effects in the form of a disordered lipid metabolism. However, several other laboratory observations, such as increased liver enzymes and a weakened renal function, contrasted this assumption. The histopathology data additionally confirmed these findings through the development of hepatic inflammation, hepatocytes’ distortion, and renal tubule’s lipid infiltration. Therefore, while Orlistat shows a promise as an anti-obesity agent, its use is impractical due to the dose-dependent hepatorenal toxicity to HFD-fed rats in a sub-chronic regimen. The above findings highlight the need for the lifelong examination of the organ function in patients undergoing persistent orlistat treatment, particularly in patients with preexisting metabolic dysfunction. Orlistat has a dose-related effect on hepatic and renal functioning. Only slight histological and functional alterations were noted at the 360 mg/kg implying protective effect to some extent. Nonetheless, the doses of 480 and 600 mg/kg could lead to severe degenerative changes of liver and kidney tissues that were an indicator of aggravated organ dysfunction. These findings support the need to optimize dosage to reduce possible hepatic and renal toxicity without affecting therapeutic effectiveness. For future directions: Elucidate the molecular mechanisms behind Orlistat -induced hepatorenal toxicity in rats, including oxidative stress, immune mediators, and apoptosis molecular markers. Investigate the gut-liver-kidney axis to determine whether the kidney and liver are damaged as a result of the direct cytotoxic effect of the medication or due to the secondary metabolic effect of drug exposure. Meanwhile, study lower doses of Orlistat to establish the therapeutic window where medication is both efficacious and safe. For future direction: Explore whether hepatorenal injury is irreversible in the HFD-fed rat model or if it can be recovered after orlistat withdrawal. Investigate the combined treatment with Orlistat and renoprotective or hepatoprotective medications to reduce the generation of toxicity.

The future studies need to be directed towards determining the long-term safety of orlistat, especially when administered on a chronic basis. In spite of the evident dose-dependent hepatorenal changes observed in the given study, it is still necessary to examine whether they continue, increase, or disappear following the drug withdrawal. Studies of long term follow-up in terms of recovery pattern, biochemical normalization and histopathological regeneration would be useful in determining the reversibility of orlistat induced toxicity. Also, the chronic-exposure models simulating the practical clinical application are required to better establish the cumulative risks of long-term therapy, particularly in the vulnerable groups.

## Declaration of generative AI use in the writing process

The authors wish to particularly note that, at the intermediate editorial stage, when this manuscript was prepared, several phrasings and wording variations were generated using a generative AI tool *Perplexity*, which targeted free of charge clarity, fluency, and readability where necessary, some of which were included in the final version of this manuscript. However, all original revisions had been selected, modified, and reviewed by the authors for accuracy and precision. The authors accept full responsibility for the final product and declare that the use of AI had no impact on the work’s results or scientific validity.

## Consent to publish

The final version of the work has been examined and approved by all authors, who also agree to its publication.

## Data Availability

The datasets generated and/or analyzed during the current study, including the completed ARRIVE 2.0 checklist, are publicly available in the Zenodo repository under the Creative Commons Zero (CC0 1.0) Public Domain Dedication. The data can be accessed at: https://doi.org/10.5281/zenodo.18225317 ( Alkadhimy et al., 2026).
