# Supplementary material for: Effect of anti-obesity drug orlistat (Xenical™) on liver and kidney function in male rats fed a high-fat diet
Source: F1000Res. 2026 Mar 31;15:136. Originally published 2026 Jan 29. [Version 2] doi: 10.12688/f1000research.174090.2 (PMC13126012; doi:10.12688/f1000research.174090.2)
Supplement: Peer review history

# Reviewer report: Reviewer response for version 2

**DOI:** 10.5256/f1000research.197366.r477543

**Published:** 2026-05-05

**Authors:** Guzide Satir-Basaran

**Affiliations:** 1 Erciyes University, Kayseri, Turkey

1. There are studies indicating that **orlistat** has harmful effects on the liver and kidneys.
2. Using **n = 5 rats per group** reduces the statistical power.
3. There is no **Normal diet + orlistat group** among the groups. This creates a major limitation in evaluation and interpretation.
4. In **Table 2**, XE-600 is mentioned—does this group correspond to ND or HFD? It is unclear. The toxicity of orlistat alone cannot be clearly distinguished, and its effect in combination with HFD is not clearly defined.
5. The doses used (360–600 mg/kg orlistat) are far above the therapeutic doses used in humans. This limits clinical applicability.
6. The decrease in **AST and ALT** creates a contradiction. Normally, these enzymes increase in liver damage. This situation has not been sufficiently explained.
7. The inflammatory markers mentioned in the study are not specified among the measured values.
8. In an obesity model, **60 days is not sufficient** to induce liver damage. Therefore, the obesity-related findings in this study are also considered inadequate.
9. Some statements in the study are contradictory. Additionally, there are issues with language and clarity.
10. Additional data supporting obesity are required for the manuscript to achieve indexing.

Is the work clearly and accurately presented and does it cite the current literature?

Partly

If applicable, is the statistical analysis and its interpretation appropriate?

No

Are all the source data underlying the results available to ensure full reproducibility?

No

Is the study design appropriate and is the work technically sound?

No

Are the conclusions drawn adequately supported by the results?

No

Are sufficient details of methods and analysis provided to allow replication by others?

No

Reviewer Expertise:

Biochemistry

I confirm that I have read this submission and believe that I have an appropriate level of expertise to state that I do not consider it to be of an acceptable scientific standard, for reasons outlined above.

# Reviewer report: Reviewer response for version 2

**DOI:** 10.5256/f1000research.197366.r477540

**Published:** 2026-04-28

**Authors:** Sabuj Kanti Nath

**Affiliations:** 1 James Cook University, Townsville, Australia

This study investigates the dose-dependent effects of orlistat on hepatic and renal function in high-fat diet-fed rats using biochemical and histopathological analyses. The topic is relevant; however, several methodological and interpretative limitations reduce the scientific strength and translational value of the study.

 The study design is only partly appropriate. While the general experimental approach is acceptable, there are important limitations, including the use of very high (supraphysiological) doses of orlistat without proper justification, a small sample size (n = 5 per group), absence of key control groups (e.g., normal diet + orlistat), and a relatively short experimental duration. These factors limit the robustness and applicability of the findings.

 The methods are described to some extent, but important details required for reproducibility are missing, particularly regarding randomization, blinding, and histopathological evaluation criteria. The statistical analysis is not sufficiently rigorous, as exact p-values, effect sizes, and detailed interpretations are lacking, and some conclusions appear inconsistent with the presented data.

 A major concern is the inconsistency between results and conclusions. The study reports improvements in lipid profile, reduced liver enzyme levels, and partial histological improvements at certain doses, yet concludes that orlistat causes severe dose-dependent hepatorenal toxicity. Additionally, the observation of decreased liver enzyme activities alongside histological evidence of hepatic damage is biologically contradictory and not adequately explained.

 Furthermore, mechanistic claims related to oxidative stress and inflammation are not supported by direct measurements and appear speculative. These should either be removed or clearly presented as hypotheses.

 To improve the manuscript, the authors should:

1. Provide clear justification for dose selection, including human-equivalent dose calculations.
2. Resolve inconsistencies between biochemical, histological, and interpretative findings.
3. Revise the conclusions to accurately reflect the results.
4. Strengthen statistical reporting with detailed outputs.
5. Improve methodological transparency (randomization, blinding, histological scoring).
6. Remove unsupported mechanistic claims.
7. Revise the manuscript for language clarity and readability.

Is the work clearly and accurately presented and does it cite the current literature?

Partly

If applicable, is the statistical analysis and its interpretation appropriate?

No

Are all the source data underlying the results available to ensure full reproducibility?

Yes

Is the study design appropriate and is the work technically sound?

Partly

Are the conclusions drawn adequately supported by the results?

Partly

Are sufficient details of methods and analysis provided to allow replication by others?

Partly

Reviewer Expertise:

Nutrigenomics, Animal Nutrition, Obesity and Metabolic Disorders, Heat Stress Biology, Feed Additives and Nutritional Interventions, Rumen Microbiology, Experimental Design in Livestock Studies

I confirm that I have read this submission and believe that I have an appropriate level of expertise to confirm that it is of an acceptable scientific standard, however I have significant reservations, as outlined above.

# Reviewer report: Reviewer response for version 2

**DOI:** 10.5256/f1000research.197366.r472108

**Published:** 2026-04-01

**Authors:** Babi Dutta

**Affiliations:** 1 Manipal Academy of Higher Education, Manipal, Karnataka, India

The authors have provided detailed feedback in response to my review comments and adequately addressed the queries raised.

Is the work clearly and accurately presented and does it cite the current literature?

Partly

If applicable, is the statistical analysis and its interpretation appropriate?

Yes

Are all the source data underlying the results available to ensure full reproducibility?

No source data required

Is the study design appropriate and is the work technically sound?

Yes

Are the conclusions drawn adequately supported by the results?

Partly

Are sufficient details of methods and analysis provided to allow replication by others?

Partly

Reviewer Expertise:

HFD induced changes, effect of herbal extracts on serum lipid profile in HFD rats

I confirm that I have read this submission and believe that I have an appropriate level of expertise to confirm that it is of an acceptable scientific standard.

# Reviewer report: Reviewer response for version 1

**DOI:** 10.5256/f1000research.191966.r458448

**Published:** 2026-03-04

**Authors:** Jan Sobczyński

**Affiliations:** 1 Medical University of Lublin, Lublin, Poland

The manuscript aims to present the toxic effects of orlistat (360, 480, 600 mg/kg, 60 days) in male rats on a high‑fat diet (HFD), reporting hematology, lipid panel, liver/kidney biochemistry, and H&E histology. While orlistat reduced obesity, it caused dose-related increases in liver enzymes and kidney damage. Structural changes in organs were more severe at higher doses of the drug. The findings conclude that orlistat administration in HFD conditions can lead to severe impairment of liver and kidney function, highlighting the need for close monitoring. The main strength of the study are: clearly stated research aim, multiangle view of the damage caused by orlistat, clear data presentation. The topic is relevant and the study provides a nuanced view into reporting of chemotherapy-related adverse effects. I would raise some points that could be better addressed by authors:

- The doses used by authors (360–600 mg/kg/day, p.o.) drastically exceed the typically used doses in animal studies which for rodent would typically include dosage of 30–40 mg/kg/day [Reference 1]; some models even 2 mg/kg for 30 days [Reference 2]. Clinically, orlistat acts locally in the gut with minimal systemic absorption, so the risk of its accumulation in the body is minimal. The manuscript provides no pharmacological or scaling rationale for supratherapeutic dosing. This undermines translational relevance and may provoke off‑target toxicity. The authors should provide a clear explanation for choosing such high doses and possible translational value of their investigation.
- There is no vehicle‑gavage control for chow and HFD groups. Orlistat groups were gavaged with an aqueous suspension; if rats belonging to control and HFD groups rats were not gavaged, procedure‑related stress may confound outcomes. The choice of not using this group should be explained.
- The number of rodents in study group (n=5/group) is underpowered for multi‑endpoint toxicology and histology, and authors did not report a priori power calculation. Please, provide a power analysis (effect size, α=0.05, 1–β≥0.8) and justify n.
- Although the authors cited a work describing HFD diet, some basic data concerning this diet should be provided for easier interpretation and classification of diet by phenotype severity (%kcal, fat content and type).
- Current literature generally reports that HFD elevates TG, LDL‑C and liver enzymes, and increases inflammatory markers, although effects vary with diet composition and the duration of diet administration to rodent. In this study HFD-fed animals are healthier than control (lower LDL-C and better liver enzymes levels). Perhaps outliers in the experiments were not properly handled or quantitation method was not accurate? Currents results make it difficult to compare and draw conclusions from treatment groups. Authors should add body weight, adiposity, and fecal fat to verify HFD phenotype and orlistat target involvement. Consider
- Some results contradict one another. Table 6 indicates severe liver damage following orlistat 600 mg/kg dosage based on pathology results. Still same dose appears to decrease ALT and AST as compared to control. This should be discussed thoroughly.
- The authors often imply that orlistat/HFD modulate oxidative stress and inflammatory cytokines (TNF‑α, IL‑6), yet no inflammatory biomarkers were measured. If you measured inflammatory markers, please show results. If this is a hypothesis, please reason with literature evidence. As similar mechanistic claims (ROS, TNF‑α/IL‑6 upregulation) are speculative without measurements. Reason with relevant literature, e.g.[Reference 3]
- Some claims need to be corrected, 1.  in Abstract: ‘Orlistat is an antihypertensive agent…’ – no, it’s anti-obesity; 2. In Methods: “desiccation” is incorrect—perhaps authors meant “dissection”
- Please, keep your manuscript tidy and consistent: use HFD+XE‑600 consistently (not XE‑600) in all tables/figures, as you use HFD+XE‑360 and HFD+XE‑480
- Some grammar polishing could be employed, e.g.: ‘In male rats given an HFD, on the histology and function of the liver and kidneys.’ Please change to ‘This study examines the dose-dependent effects of orlistat on the histology and function of the liver and kidneys in male rats given an HFD’
- Most literature is 5 years or older, some references are from the last century. Some newer literature should be considered for citations, even if quantitation methods are well established.

Is the work clearly and accurately presented and does it cite the current literature?

Partly

If applicable, is the statistical analysis and its interpretation appropriate?

Partly

Are all the source data underlying the results available to ensure full reproducibility?

Yes

Is the study design appropriate and is the work technically sound?

Yes

Are the conclusions drawn adequately supported by the results?

No

Are sufficient details of methods and analysis provided to allow replication by others?

Yes

Reviewer Expertise:

effect of high fat diet on metabolism

I confirm that I have read this submission and believe that I have an appropriate level of expertise to confirm that it is of an acceptable scientific standard, however I have significant reservations, as outlined above.

## References

[1] : Comparison of Efficacy of Fermented Garlic and Orlistat (Lipase Inhibitor) in Obesity Management Using an Experimental Rodent Model. *Foods* .2023;12(21) : 10.3390/foods12213905 10.3390/foods12213905 PMC1064777837959027

[2] : Orlistat and Metformin Effectively Reduce Pancreatic Dysfunction, Insulin Resistance and Blood Glucose Levels in Male Rats Experimentally Induced with Obesity. *International Journal of Pharmacology* .2024;20(5) : 10.3923/ijp.2024.726.734 726-734 10.3923/ijp.2024.726.734

[3] : Long Feeding High-Fat Diet Induces Hypothalamic Oxidative Stress and Inflammation, and Prolonged Hypothalamic AMPK Activation in Rat Animal Model. *Frontiers in Physiology* .2018;9: 10.3389/fphys.2018.00818 10.3389/fphys.2018.00818 30034345 PMC6043859

# Reviewer report: Reviewer response for version 1

**DOI:** 10.5256/f1000research.191966.r458447

**Published:** 2026-02-28

**Authors:** Babi Dutta

**Affiliations:** 1 Manipal Academy of Higher Education, Manipal, Karnataka, India

1. Introduction (Line 6) Rinella et al 2023. Reference is about new fatty liver disease nomenclature. Modify the reference

 2. Introduction (Line 12) Dludla et al 2018. This reference does not include orlistat as good treatment, it talks about gallic acid treatment

 3. Introduction (line 16-17). Obesity is caused by these disorders, or obesity increases the risk of these disorders (rewrite for clarity)

 4. Orlistat reduces dietary fat absorption by 30% (Is there any reference for the reduction by 30% statement)

 5. Orlistat has less than 1% bioavailability and scarcely produce systemic toxicity (Reference Al-Sabah the economic impact of obesity in Kuwait: a micro-costing study evaluating the burden of obesity related comorbidities), Check the reference clearly

 6. For orlistat dosage: 360 mg/kg dosage how was the solution prepared, three tablet contents in how much ml of water to prepare stock solution of what concentration/ml for oral gavage? Reproducibility may be difficult since it is not clearly mentioned.

 7. Experimental animals (line 10). The rats were put to sleep with isoflurane and killed after 60 days. Following their desiccation, (anesthetized is a better word, desiccation meaning?)

 8.  "Anesthesia of the rat was induced with inhaled isoflurane" this section should be before the previous section "putting the rats to sleep", since it explains about blood collection after sacrifice. 

 9. Table 2 onwards HFD+XE-600 is written as XE-600, maintain uniformity

 10. After Table 4, 5th line.. HF-diet or KF diet? what is KF diet? Rectify 

 11. Explain why hepatic enzymes are reduced in HFD group than normal controls? What is meant by adaptive metabolic reactions or model distinctions? since hepatic histopathology shows steatotic changes, explain the reason why it is not reflected in liver enzyme serum profile?

 12. Liver histopathological pictures at what magnifcation is not mentioned.

 ** mark is not visible in the picture

 Curved arrows are not clearly visible

 what about 480 mg/kg dosage, no mention of that.. the effect of 480mg/kg dosage on hepatic histology is missing? 

 13. After Figure 5 of liver histopathology, HFD+XE-480 effects explanation missing?

 14. Orlistat hepatoprotective effect is probably due to (After image 5 line no 15 onwards) Contradictory statements since earlier the authors have written orlistat may cause oxidative stress caused by overproduction of reactive oxygen species (ROS), which results in lipid peroxidation and degradation of

 antioxidant defense mechanisms SOD, CAT, and GSH. This may cause increase of liver enzymes and structural hepatic degeneration that was observed in the treated groups. Also, orlistat is said to cause inflammatory reactions by upregulating pro-inflammatory cytokines (e.g., TNF- a, IL- 6), which add to the aggravation of hepatocellular and renal damages (contradictory statements)

 14. Kidney histology magnification not mentioned, every image should be from same set of area in the kidney, for example, comparison of glomerular section across the groups at same magnification. 

 15. Figure 7A is completely distorted. Provide a better picture

 16. Figure 8B shows a lot of vacuoles and epithelial damage, so how is it relatively normal. explain

 17. Image 9 and 10 picture shows renal tissue with orlistat dose of 480 and 600 and denotes renal pathology is almost normal, so why overt renal toxicity at high dose in table 6. Justify

 18. Table 6: Explanation of XE-480 dosage Figure 9. Rats fed a high-fat diet (HFD) and treated with Orlistat (XE-480) appear mostly normal, although there might be some slight changes in the structure of the renal tubules,suggesting a possible mild effect of the high-fat diet that is being mitigated by the dose of XE.

 19. Table 6: Explanation of XE-600 dosage Figure 10. Rats fed a high-fat diet (HFD) and treated with Orlistat (XE-600) appear mostly normal... So is it damaged or not.. if damage, the pictures do not justify damage

Is the work clearly and accurately presented and does it cite the current literature?

Partly

If applicable, is the statistical analysis and its interpretation appropriate?

Yes

Are all the source data underlying the results available to ensure full reproducibility?

No source data required

Is the study design appropriate and is the work technically sound?

Yes

Are the conclusions drawn adequately supported by the results?

Partly

Are sufficient details of methods and analysis provided to allow replication by others?

Partly

Reviewer Expertise:

HFD induced changes, effect of herbal extracts on serum lipid profile in HFD rats

I confirm that I have read this submission and believe that I have an appropriate level of expertise to confirm that it is of an acceptable scientific standard, however I have significant reservations, as outlined above.
